# Childhood maltreatment experiences are associated with altered diffusion in occipito‐temporal white matter pathways

**DOI:** 10.1002/brb3.1485

**Published:** 2019-11-26

**Authors:** Elizabeth A. Olson, Tate A. Overbey, Caroline G. Ostrand, Diego A. Pizzagalli, Scott L. Rauch, Isabelle M. Rosso

**Affiliations:** ^1^ Center for Depression, Anxiety and Stress Research McLean Hospital Belmont Massachusetts; ^2^ Department of Psychiatry Harvard Medical School Boston Massachusetts; ^3^ McLean Imaging Center McLean Hospital Belmont Massachusetts

**Keywords:** childhood maltreatment, diffusion tensor imaging, inferior longitudinal fasciculus, posttraumatic stress disorder, probabilistic tractography

## Abstract

**Background:**

Childhood maltreatment may contribute to brain alterations in posttraumatic stress disorder (PTSD). We previously found that PTSD was associated with white matter compromise, or lower fractional anisotropy (FA), in the left inferior longitudinal fasciculus (ILF). In this study, including non‐PTSD controls, we examined whether ILF FA was associated with maltreatment exposures, including those that meet DSM‐IV criterion A (physical abuse, sexual abuse) and those that typically do not (emotional abuse, emotional neglect, physical neglect). We hypothesized that lower FA would be associated with PTSD diagnosis and with both categories of maltreatment.

**Methods:**

Ninety‐three participants (51 female), ages 20–50, were enrolled, including 32 with lifetime DSM‐IV PTSD, 27 trauma‐exposed non‐PTSD controls, and 34 healthy controls. Participants completed structured interviews, the Childhood Trauma Questionnaire (CTQ), and diffusion‐weighted imaging (36 directions). Probabilistic tractography (using FreeSurfer's TRACULA) was used to assess diffusion metrics in the ILF.

**Results:**

Contrary to our hypothesis, there was no significant effect of diagnostic group on FA. In contrast, higher CTQ scores were significantly associated with lower FA in the ILF bilaterally. This association of maltreatment with lower FA remained statistically significant after controlling for diagnostic group, and it was significant for both criterion‐A‐type and noncriterion‐A‐type maltreatment categories.

**Conclusions:**

This work contributes to a growing body of literature indicating that different forms of childhood maltreatment are associated with altered white matter microstructure in the ILF, an association pathway involved in integrating visual information from occipital regions with emotion processing functions of the anterior temporal lobe.

## Graphical abstract

Childhood maltreatment, but not posttraumatic stress disorder (PTSD) diagnostic status, is associated with altered diffusion in occipito‐temporal white matter pathways including lower fractional anisotropy in the inferior longitudinal fasciculus. This work contributes to a growing body of literature indicating that different forms of childhood maltreatment are associated with altered white matter microstructure in the ILF, an association pathway involved in integrating visual information from occipital regions with emotion processing functions of the anterior temporal lobe.

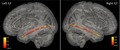

## 1 INTRODUCTION

Childhood maltreatment is associated with a broad range of negative developmental outcomes, including socioemotional, behavioral, and attachment problems (Cicchetti & Toth, 2005). Maltreated children have higher risk of adverse psychiatric outcomes (Kessler et al., 2010), including posttraumatic stress disorder (PTSD; Widom, 1999). This association likely reflects complex causal relationships. First, childhood maltreatment may lead to PTSD as a direct response to the exposure; prospectively ascertained maltreatment exposure is indeed associated with a markedly increased rate of PTSD (Scott, Smith, & Ellis, 2010). Second, childhood maltreatment may increase risk for the development of PTSD in response to adult trauma (Breslau et al., 2014). From a neurobiological perspective, childhood maltreatment has been associated with physiological processes including chronic inflammation (Coelho, Viola, Walss‐Bass, Brietzke, & Grassi‐Oliveira, 2014) and altered hypothalamic–pituitary–adrenal (HPA) axis reactivity (Heim, Newport, Mletzko, Miller, & Nemeroff, 2008; Suzuki, Poon, Papadopoulos, Kumari, & Cleare, 2014), which may increase the risk for aberrant stress responses and PTSD following subsequent trauma exposure (Carvalho, Coimbra, Ota, Mello, & Belangero, 2017; Eraly et al., 2014). Similarly, childhood maltreatment can disrupt the development of social‐emotional capacities such as attachment (Baer & Martinez, 2006) and social interconnectedness (Hovens, Giltay, van Hemert, & Penninx, 2016), thereby increasing the likelihood of PTSD following subsequent trauma exposure (Lee & Youm, 2011; Platt, Keyes, & Koenen, 2014; Sippel et al., 2017). Collectively, this literature suggests that childhood maltreatment moderates the neural expression of PTSD (Teicher & Samson, 2013).

Previously, we examined 10 white matter (WM) tracts in participants with PTSD versus trauma‐exposed controls, including those connecting canonical fear circuitry regions (e.g., the uncinate fasciculus, UF), and found lower fractional anisotropy (FA) and higher radial diffusivity (RD) in the left inferior longitudinal fasciculus (ILF; Olson et al., 2017). FA and RD, along with mean diffusivity (MD) and axial diffusivity (AD) are diffusion metrics that assess the extent of directionality of water diffusion and reflect brain structural organization. The ILF is a visual limbic pathway that projects between the occipital and anterior temporal lobes, facilitating rapid transfer of visual information to and from temporal regions involved in memory, learning, and emotional processing (Catani, Jones, Donato, & Ffytche, 2003). Choi, Jeong, Polcari, Rohan, and Teicher (2012) also reported lower FA in the left ILF of individuals who witnessed parental domestic violence (WDV) early in life. Our finding was the first report of abnormalities in the ILF in association with PTSD. Because the trauma‐exposed non‐PTSD participants in our sample endorsed minimal exposure to childhood maltreatment, we could not statistically separate effects of childhood maltreatment from effects of PTSD diagnostic status. One of the overarching goals of the current study was to address this limitation in a sample exposed to diverse forms of maltreatment.

One distinction between research on PTSD and research on childhood maltreatment relates to the types of index events under study. PTSD requires exposure to criterion A traumatic event(s) that involve “actual or threatened death or serious injury, or a threat to the physical integrity of self or others,” including “violent personal assault (sexual assault, physical attack).” Physical abuse and sexual abuse typically meet criterion A, while other forms of maltreatment, including emotional abuse, emotional neglect, and physical neglect, typically do not. A growing body of literature suggests that exposure to forms of childhood maltreatment that do not meet criterion A is at least as detrimental as exposure to forms of maltreatment that do meet criterion A. For example, Khan et al. (2015) found that adolescent exposure to verbal abuse, emotional neglect, and nonverbal emotional abuse was more strongly associated with history of major depressive disorder than physical or sexual abuse. Additionally, brain imaging studies have shown that noncriterion‐A‐type maltreatment experiences have significant neurobiological consequences. Specifically, neglect and emotional abuse are associated with amygdala and hippocampus hyperactivity in response to negative emotion (Bogdan, Williamson, & Hariri, 2012; Lee et al., 2015; Maheu et al., 2010), and with amygdala–hippocampus hyperconnectivity in response to psychosocial stress (Fan et al., 2015). Emotional neglect may be uniquely detrimental to social development; in a sample of maltreated adolescents, while controlling for other forms of maltreatment, only emotional neglect independently predicted antisocial features (Ometto et al., 2016).

Directly relevant to the current analyses, the neural correlates of maltreatment and posttraumatic psychopathology overlap substantially. Early life stress is associated with amygdala hyperactivity when viewing negatively valenced facial expressions and with lower hippocampal volume (Dannlowski et al., 2012; Rao et al., 2010; Teicher, Anderson, & Polcari, 2012; Vythilingam et al., 2002), both of which are also robust findings in PTSD patients (Bromis, Calem, Reinders, Williams, & Kempton, 2018; Etkin & Wager, 2007; Hayes, Hayes, & Mikedis, 2012; Smith, 2005). Additionally, both childhood maltreatment and PTSD are associated with alterations in white matter (WM) structural integrity. Tractography studies in PTSD samples versus trauma‐exposed controls have identified significantly lower FA and/or higher mean diffusivity (MD) in fronto‐temporal WM tracts including the UF (Koch et al., 2017), genu of the corpus callosum (Sun et al., 2013), and cingulum (Fani et al., 2016; Reuveni et al., 2016). In the childhood maltreatment literature, many prior reports have focused on reduced FA in the UF, a tract frequently selected for a priori analysis because of its role in facilitating prefrontal regulation of amygdala activity (e.g., Eluvathingal et al., 2006; Hanson, Knodt, Brigidi, & Hariri, 2015; Ho et al., 2017; Souza‐Queiroz et al., 2016). While the UF is often selected as an a priori target, altered diffusion in other tracts also has been identified in the context of diverse types of early adverse experiences, ranging from verbal abuse to early institutionalization, in samples with and without psychopathology. For example, Huang, Gundapuneedi, and Rao (2012) found lower FA in the superior longitudinal fasciculus (SLF), cingulum, inferior fronto‐occipital fasciculus, and splenium of the corpus callosum in adolescents who had experienced physical abuse, sexual abuse, or witnessed domestic violence. In young adults without psychopathology but with prior exposure to chronic parental verbal abuse, Choi, Jeong, Rohan, Polcari, and Teicher (2009) found lower FA in the arcuate fasciculus, cingulum, and fornix. In a sample exposed to early institutional care, Hanson et al. (2013) found widespread alterations in FA that were particularly extensive in posterior cortical regions. Finally, abnormal diffusion in posterior portions of the corpus callosum has been reported in children with maltreatment‐related PTSD (De Bellis et al., 2015; Jackowski et al., 2008). In addition to these cross‐sectional findings, longitudinal studies have examined baseline diffusion as a predictor of the development of psychopathology in maltreated samples, with increased internalizing symptoms developing over time in the context of lower baseline FA in the UF (Hanson et al., 2015), SLF and cingulum (Huang et al., 2012), and corpus callosum and external capsule (Bick, Fox, Zeanah, & Nelson, 2017). Thus, the PTSD and childhood maltreatment tractography literatures both implicate fronto‐temporal white matter tracts, though significant findings extend beyond those fibers to include more posterior cortical regions.

To summarize, the goal of the present study was to extend our previous finding of lower ILF FA in PTSD in a new sample that included comparison subjects with reported exposure to a broad range of childhood maltreatment severity, in order to dissociate possible effects of PTSD versus childhood maltreatment on white matter microstructure. We hypothesized that lower ILF FA would be associated with both PTSD diagnosis and exposure to childhood maltreatment. Additionally, because of the demonstrated significance of emotional neglect, emotional abuse, and physical neglect on neurobiological development, we hypothesized that noncriterion‐A‐type maltreatment exposures would have comparable associations with ILF FA as criterion‐A‐type maltreatment exposures. As a control tract, we examined the corticospinal tract (CST), a pyramidal tract involved in motor control. We were unable to locate any prior reports of altered FA in the CST in individuals with PTSD or histories of childhood maltreatment.

## 2 MATERIALS AND METHODS

### 2.1 Participants

Ninety‐three subjects who responded to study advertisements in the Boston metropolitan area participated in this study, including 32 participants with DSM‐IV lifetime PTSD, 27 trauma‐exposed non‐PTSD controls (TENC), and 34 healthy controls (HC). Prior to participating, all subjects received a full explanation of study procedures and provided written informed consent. All subjects were paid for their time ($25/hr, up to $200). Study procedures were authorized by Partners HealthCare's Human Research Committee. For detailed inclusion/exclusion criteria, see Appendix S1.

### 2.2 Clinical interviews and measures

Participants were evaluated with the Structural Clinical Interview for DSM‐IV Axis I Disorders (SCID‐I/P) (First, Spitzer, Gibbon, & Williams, 1996). PTSD symptom severity was assessed using the Clinician‐Administered PTSD Scale, Current and Lifetime Versions (CAPS‐DX) (Blake et al., 1995). The CAPS was administered to all participants who endorsed at least one DSM‐IV criterion A event on the Life Event Checklist (Gray, Litz, Hsu, & Lombardo, 2004). Clinical interviews were conducted by doctoral‐level clinical psychologists. Lifetime trauma load was assessed using the Life Events Checklist (LEC: Gray et al., 2004); the total score was the sum of experienced and witnessed events).

Childhood Trauma Questionnaire (Bernstein et al., 2003) subscales were summed to generate a total maltreatment exposure variable. The physical and sexual abuse subscales were summed in order to create a combined criterion‐A‐type maltreatment exposure score; and the emotional neglect, physical neglect, and emotional abuse subscales were summed to create a noncriterion‐A‐type maltreatment exposure score.

### 2.3 MR image acquisition

MRI data were acquired via a 3.0 Tesla Siemens Tim Trio Scanner (Siemens) with the use of a 32‐channel phased‐array design RF head coil operating at 123 MHz. Anatomical whole‐brain images were obtained, including high‐contrast T1‐weighted MPRAGE images. Whole‐brain diffusion tensor images (DTI) were obtained using a 36 direction‐weighting scheme. (For parameters, see Appendix S1.)

### 2.4 Tractography processing

Automatic probabilistic tractography was performed using TRActs Constrained by UnderLying Anatomy (TRACULA, v 1.22.2.12) tool (Yendiki et al., 2011) within FreeSurfer 5.3.0, using a standard pipeline (see Appendix S1). FA, MD, RD, and axial diffusivity (AD) from each tract (ILF, CST) were extracted for each participant. A modified total motion index was calculated following Yendiki, Koldewyn, Kakunoori, Kanwisher, and Fischl (2014).

### 2.5 Statistical analyses

Statistical analyses were performed using SPSS Statistics 20 (IBM Corporation). Because of violations of the assumption of homogeneity of variances, group differences in maltreatment exposure were evaluated using Welch's ANOVA with Games‐Howell post hoc tests. Multivariate analyses examined the effect of diagnostic group (HC, TENC, PTSD) and childhood maltreatment exposure on FA, with right and left ILF as dependent variables. Because age and sex can affect FA, these variables were included as nuisance covariates in all analyses. FA was treated as the primary measure of interest; significant findings for FA were followed by analyses of other diffusion metrics (MD, RD, AD) to further characterize changes in the diffusion signal. FA captures the extent to which diffusion occurs preferentially along one direction, AD assesses diffusion along the long axis and is primarily influenced by changes in axonal integrity, RD assesses diffusion perpendicular to the long axis and is primarily influenced by alterations in myelination, and MD captures the overall extent of diffusion restriction (Winklewski et al., 2018).

## 3 RESULTS

### 3.1 Childhood maltreatment exposure

**Figure 1:**
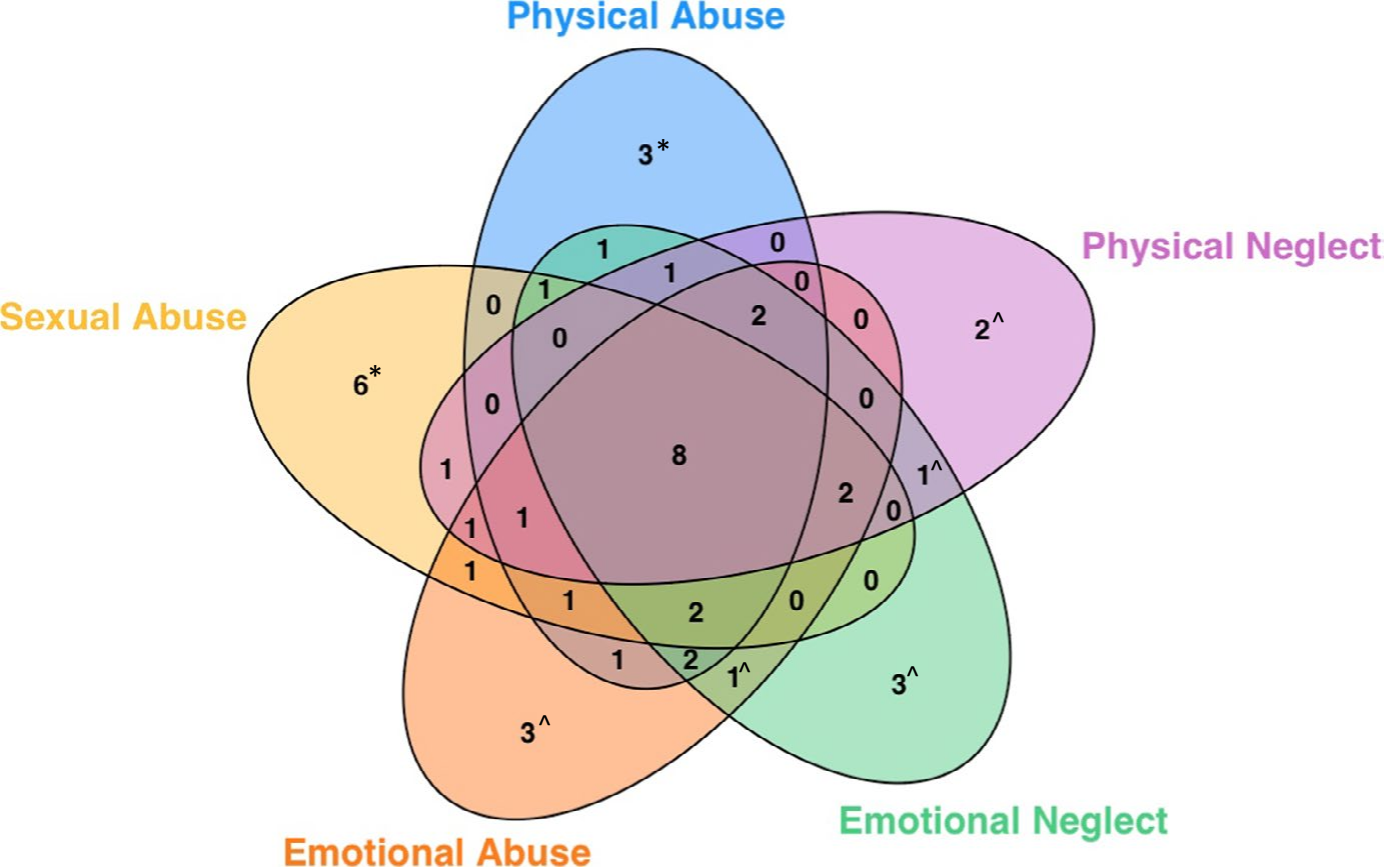
Maltreatment exposures in this sample. Forty‐three of 93 participants endorsed at least moderate maltreatment exposure. As illustrated here, maltreatment exposure categories were largely overlapping. Numbers reflect number of participants reporting exposure to the indicated maltreatment types. Numbers with carets reflect individuals exposed only to noncriterion‐A‐type maltreatment types, while numbers with asterisks reflect individuals exposed to only criterion‐A‐type maltreatment types. All others reported exposure to both maltreatment categories

Demographics and clinical characteristics of the sample are presented in Table 1. When maltreatment was coded as present or absent based on criteria for at least moderate exposure, 49 participants reported no maltreatment exposure, while 43 participants endorsed at least one category of moderate or severe maltreatment exposure (one participant did not provide complete data). Exposure to maltreatment types was largely overlapping (Figure 1): participants exposed to one maltreatment type tended to be exposed to other maltreatment types as well. Nine participants endorsed exposure only to criterion‐A‐type maltreatment (physical or sexual abuse), and 10 participants endorsed exposure only to noncriterion‐A‐type maltreatment (emotional abuse, emotional neglect, or physical neglect). Independent samples *t* tests demonstrated no sex differences in the severity of maltreatment exposure, except for sexual abuse, which was more severe in females (*M* = 8.78, *SD* = 5.89) than in males (*M* = 6.32, *SD* = 3.74), *t*(85.79) = −2.44, *p* = .02.

**Table 1:** Demographic and clinical characteristics of posttraumatic stress disorder (PTSD), trauma‐exposed non‐PTSD control (TENC), and healthy control (HC) participants (mean ± *SD* or *N *(%))

	PTSD *N* = 32	TENC *N* = 27	HC *N* = 34	Group differences
Sex (female)	19 (59%)	15 (56%)	17 (50%)	*X* ^2^(2) = 0.59, *p*=.74
Age	34.87 ± 8.11	29.99 ± 7.04^a^	35.00 ± 8.96	*F*(2, 90) = 3.53, *p* = .03
CAPS current total	41.59 ± 23.38	8.44 ± 10.30	—	*t*(44.11) = −7.23, *p* < .001
Total motion index	0.49 ± 1.10	−0.18 ± 1.49	0.30 ± 1.65	*F*(2, 90) = 1.64, *p* = .20
CTQ, Emotional abuse	12.78 ± 6.69	9.85 ± 4.29	6.71 ± 2.70	*F*(2, 50.63) = 14.39, *p* < .001^b^
CTQ, Physical abuse	9.66 ± 5.04	7.96 ± 3.63	5.82 ± 2.30	*F*(2, 51.37) = 9.46, *p* < .001^b^
CTQ, Sexual abuse	10.25 ± 6.69	7.58 ± 4.76	5.35 ± 1.61	*F*(2, 41.79) = 10.02, *p* < .001^c^
CTQ, Emotional neglect	14.66 ± 5.43	11.48 ± 4.78	8.21 ± 3.59	*F*(2, 55.33) = 16.67, *p* < .001^b^
CTQ, Physical neglect	9.28 ± 3.74	6.81 ± 2.17	5.65 ± 1.30	*F*(2, 49.34) = 14.85, *p* < .001^d^
Criterion‐A‐type maltreatment	19.91 ± 10.42	15.46 ± 7.30	11.18 ± 3.36	*F*(2, 45.13) = 12.65, *p* < .001^b^
Noncriterion‐A‐type maltreatment	36.72 ± 14.09	28.15 ± 10.15	20.56 ± 6.54	*F*(2, 51.65) = 19.67, *p* < .001^d^
Total CTQ score	56.63 ± 22.11	43.11 ± 16.12	31.74 ± 9.38	*F*(2, 48.83) = 19.55, *p* < .001^d^
Number of participants taking psychotropic medication	2 (6%)	1 (4%)	0 (0%)	
Lifetime trauma load (LEC: experienced plus witnessed events)	8.41 ± 4.63	6.48 ± 2.85	2.56 ± 1.94	*F*(2, 51.74) = 33.54, *p* < .001^b^

Abbreviations: CAPS, Clinician‐Administered PTSD Scale; CTQ, Childhood Trauma Questionnaire.

a TENC < HC, TENC < PTSD.

b HC < TENC, HC < PTSD.

c HC < PTSD.

d HC < TENC < PTSD.

### 3.2 ILF FA: Associations with PTSD diagnosis and total childhood maltreatment severity

**Figure 2:**
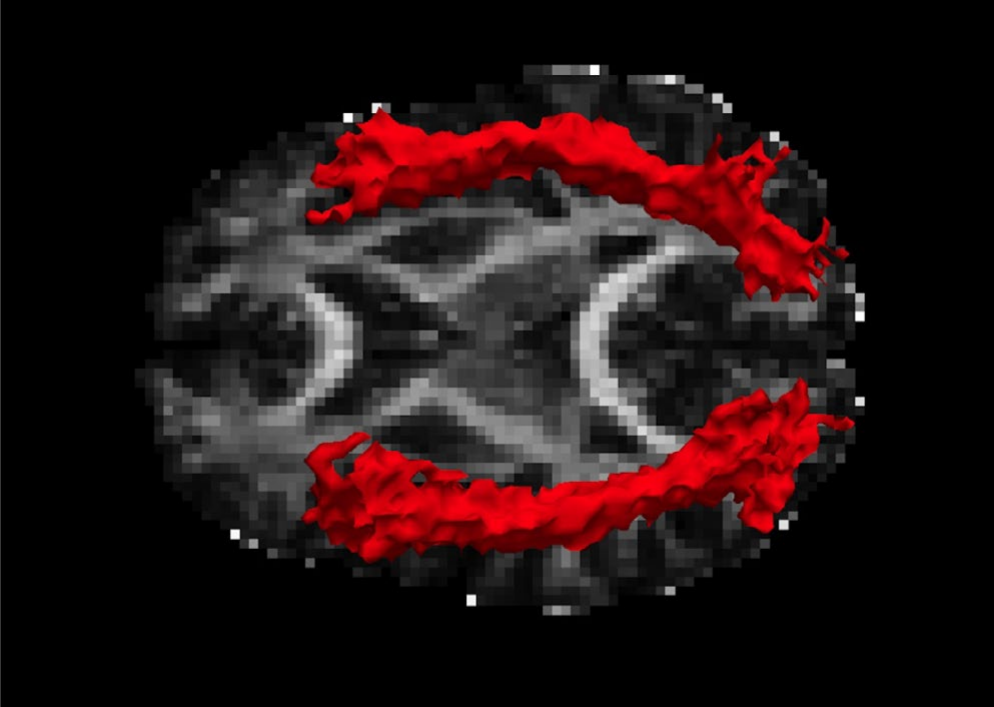
Tractography of the inferior longitudinal fasciculus in one representative case

Contrary to our hypotheses, there was no significant effect of diagnostic group on ILF FA, *F*(4, 176) = 1.11, *p* = .36, partial eta squared = 0.03 (Figure 2). In contrast, greater total childhood maltreatment severity was associated with lower ILF FA, *F*(2, 87) = 3.66, *p* = .03, partial eta squared = 0.08. This effect was significant bilaterally (left ILF, *F*(1, 88) = 6.79, *p* = .01; right ILF, *F*(1, 88) = 5.35, *p* = .02). The relationship between childhood maltreatment and lower ILF FA persisted in the combined sample after controlling for diagnostic group, *F*(2, 85) = 4.12, *p* = .02, and after controlling for lifetime trauma load, *F*(2, 86) = 3.233, *p* = .044, which was not significantly associated with ILF FA, *F*(2, 86) = 0.140, *p* = .870. Total childhood maltreatment was also significantly associated with lower ILF FA within the combined TENC and PTSD groups (i.e., after excluding the HC participants), *F*(2, 53) = 3.55, *p* = .04 (see also Table 2). There were no significant associations between childhood maltreatment and the other diffusion metrics (MD, RD, AD).

**Table 2:** Partial correlations between scores on Childhood Trauma Questionnaire (CTQ) subscales and right and left ILF FA, controlling for sex and age. Lower portion of table: in combined three‐group sample (*n* = 92–93). Upper portion of table: in trauma‐exposed sample (*n* = 58–59)

Measure	1	2	3	4	5	6	7	8	9
1. CTQ emotional abuse	—	0.757*	0.481*	0.772*	0.602*	0.679*	0.918*	−0.301*	−0.293*
2. CTQ physical abuse	0.795*	—	0.578*	0.662*	0.479*	0.862*	0.733*	−0.352*	−0.213
3. CTQ sexual abuse	0.558*	0.623*	—	0.419*	0.325*	0.912*	0.470*	−0.240	−0.145
4. CTQ emotional neglect	0.796*	0.706*	0.513*	—	0.683*	0.594*	0.931*	−0.275*	−0.169
5. CTQ physical neglect	0.672*	0.571*	0.446*	0.723*	—	0.443*	0.807*	−0.334*	−0.315*
6. CTQ criterion‐A‐type maltreatment	0.737*	0.880*	0.920*	0.666*	0.557*	—	0.660*	**−0.330** *	**−0.197**
7. CTQ noncriterion‐A‐type maltreatment	0.929*	0.779*	0.565*	0.941*	0.836*	0.773*	—	**−0.333** *	**−0.281** *
8. Right ILF FA	−0.242*	−0.261*	−0.201	−0.164	−0.257*	**−0.250** *	**−0.236** *	—	0.660*
9. Left ILF FA	−0.280*	−0.223*	−0.197	−0.159	−0.288*	**−0.232** *	**−0.257** *	0.653*	—

Note

Bold: correlations between broad maltreatment measures and ILF FA.

Abbreviations: FA, fractional anisotropy; ILF, inferior longitudinal fasciculus.

* *p* < .05.

### 3.3 ILF FA: Relationships with childhood maltreatment types

**Figure 3:**
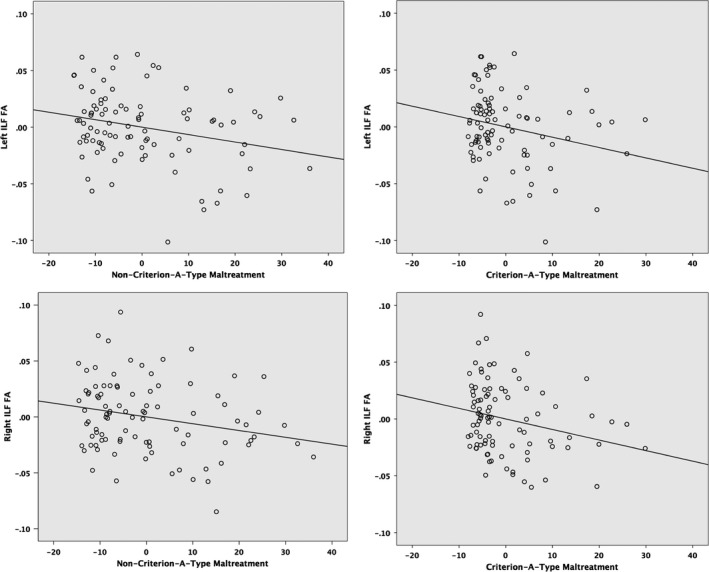
Scatter plots of partial regressions of childhood maltreatment on inferior longitudinal fasciculus (ILF) fractional anisotropy (FA), controlling for age and sex. Left, association between noncriterion‐A‐type maltreatment (physical neglect, emotional neglect, emotional abuse) and left (top) and right (bottom) ILF FA. Right, association between criterion‐A‐type maltreatment (physical and sexual abuse) and left (top) and right (bottom) ILF

The association between childhood maltreatment severity and ILF FA was significant for both criterion‐A‐type maltreatment exposures (i.e., physical and sexual abuse), *F*(2, 87) = 3.15, *p* = .048, and for noncriterion‐A‐type maltreatment exposures (i.e., emotional abuse, and emotional and physical neglect), *F*(2, 88) = 3.52, *p* = .03 (Figure 3). The relationship of noncriterion‐A‐type maltreatment exposures with bilateral ILF FA persisted after controlling for diagnostic group, *F*(2, 86) = 3.91, *p* = .02, with a nonsignificant effect of diagnostic status, *F*(4, 174) = 1.33, *p* = .26. Similarly, the association of criterion‐A‐type maltreatment exposures with bilateral ILF FA remained after controlling for diagnostic group, *F*(2, 85) = 3.14, *p* = .048, with a nonsignificant effect of diagnostic status, *F*(4, 172) = 1.78, *p* = .14.

### 3.4 ILF FA: Associations of childhood maltreatment along the trajectory of the ILF pathway

**Figure 4:**
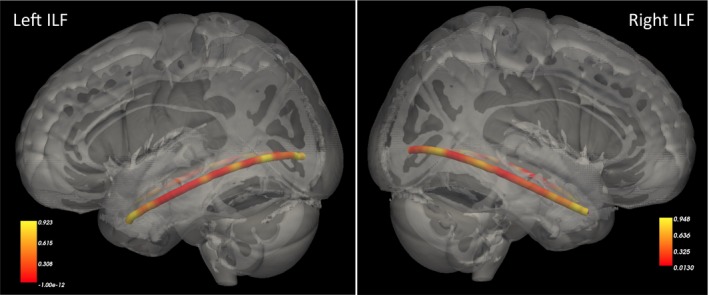
Heat maps illustrating *p*‐values for the association between total childhood maltreatment (Childhood Trauma Questionnaire total score) and fractional anisotropy along the trajectory of the inferior longitudinal fasciculus

To examine relationships with diffusivity along the trajectory of the ILF pathway, individual results were projected into MNI space along the tract, and *p*‐values at each point along the normalized tract were computed for the association between total childhood maltreatment exposure and FA (at 53 points along the left ILF, and 52 points along the right ILF; Figure 4). In the left hemisphere, there were extensive regions along the trajectory of the ILF where FA was significantly associated with childhood maltreatment. These ran in parallel with much of the hippocampus, ranging from regions near the anterior hippocampus to regions near the posterior hippocampus and posterior parahippocampal gyrus. Additionally, there was one left hemisphere region near the fusiform gyrus that showed a significant relationship with childhood maltreatment. In the right hemisphere, the findings were somewhat more localized, with significant associations of maltreatment with FA in the right temporal lobe near the mid‐hippocampus, in the posterior temporal lobe near the lingual gyrus, and near the occipital fusiform gyrus.

### 3.5 CST FA: Control tract associations with PTSD diagnosis and total childhood maltreatment severity

Highlighting the specificity of the ILF findings, there was no significant effect of diagnostic group on CST FA, *F*(4, 176) = 0.69, *p* = .60, nor a significant effect of total childhood maltreatment on CST FA, *F*(2, 87) = 0.44, *p* = .65.

## 4 DISCUSSION

Greater childhood maltreatment was associated with lower FA in the ILF bilaterally, an effect that persisted after accounting for diagnostic group. In contrast, there were no significant differences in ILF FA between diagnostic groups (PTSD vs. TENC vs. healthy control). Lower ILF FA emerged in association with both criterion‐A‐type childhood maltreatment (physical and sexual abuse) and noncriterion‐A‐type maltreatment (emotional abuse and emotional and physical neglect). There were no significant relationships with other diffusion metrics (MD, RD, AD), and there were no significant associations of childhood maltreatment or PTSD diagnostic status with WM integrity of the corticospinal tract, which was selected as a control tract.

In our previous report (Olson et al., 2017), we found lower ILF FA in adults with PTSD but were unable to determine whether this reflected an association with PTSD diagnosis versus childhood maltreatment because these two variables were statistically overlapping. The present analyses (a) confirm involvement of lower WM integrity in the ILF and (b) clarify that this effect tracks with childhood maltreatment and not PTSD diagnosis. Our findings align with previous research from Choi et al. (2012), who described lower FA in the ILF of young adults who had witnessed domestic violence as children compared with young adults with no maltreatment history. Together, these results confirm that structural integrity of the ILF may be compromised in individuals who experience childhood maltreatment, with effects evident for multiple types of maltreatment. The present study found significant effects on FA but not other diffusion metrics; this pattern can be generated in the context of modest changes in RD and AD that combine to produce significant changes in the extent of directional diffusion, reflecting possible contributions of processes related to both myelination and axonal integrity. Our results contribute to a growing body of work highlighting the importance of considering posterior cortical regions in addition to canonical pathways (e.g., fronto‐temporal pathways) when evaluating effects of maltreatment and trauma exposure (De Bellis et al., 2015; Olson, Kaiser, Pizzagalli, Rauch, & Rosso, 2018).

Although the present study does not allow for differentiation of subcomponents of the ILF, anatomical work has demonstrated that this tract includes three components (fusiform, lingual, and dorsolateral‐occipital), all of which terminate in the anterior or middle temporal lobes (Latini et al., 2017). The ILF directly contacts other association tracts, including the uncinate and arcuate fasciculi (Herbet, Zemmoura, & Duffau, 2018). Like all WM tracts, the developmental trajectory of the ILF is protracted, with peak FA reached after age 25; however, other tracts that have more often been the focus of trauma research show a much later peak FA age (e.g., mid‐40s for the cingulum, over age 35 for the uncinate) (Lebel et al., 2012). It is possible that the earlier maturational process in the ILF makes it particularly vulnerable to insult in childhood or adolescence, since actively maturing regions may be more susceptible to disruption (Rice & Barone, 2000). From a functional perspective, the ILF is involved in a variety of functions including object recognition, facial recognition, and reading (Herbet et al., 2018), as well as semantic memory (Hodgetts et al., 2017). Hanson et al. (2013) found that reduced ILF FA in children exposed to emotional neglect via institutional rearing was correlated with deficits in visual paired associates learning. Importantly, ILF disruption also is associated with deficits in processing facial affect (Genova et al., 2015; Rigon, Voss, Turkstra, Mutlu, & Duff, 2018). The latter may be particularly relevant to children who have experienced maltreatment, who may experience a cascade in which maltreatment results in damage to facial affect processing centers, placing them at risk for further adverse social experiences.

Given the cross‐sectional nature of our data, it is not possible to evaluate the nature of the causal relationships between low ILF FA and maltreatment. There are a number of possibilities. First, stress exposure during childhood may alter ILF development, for example through mechanisms such as alterations in HPA axis responsivity, perhaps mediated by amygdala hyperactivity. Second, ILF FA reductions may occur in the context of neurobiological responses to disrupted attachment. Nonthreat‐based maltreatment including neglect can result in broad disruption to neurodevelopment extending beyond limbic regions (McLaughlin, Sheridan, & Nelson, 2017). Third, in instances where the maltreatment experience is generated by the biological parent, the interpretation of these effects is particularly complex. For example, parents who engage in abnormal attachment‐related behaviors such as emotional neglect may influence the child's neurobiological outcome through the delivery of that maltreatment experience, and also through shared neurobiology (South, Schafer & Ferraro, 2015). Further work with longitudinal imaging throughout childhood, family studies, and studies examining neurocognitive functions supported by the ILF will be necessary in order to disentangle these potential causal influences.

This study has several limitations. First, given the cross‐sectional nature of our data, we are not able to delineate causal relationships. Second, research evaluating effects of PTSD and childhood maltreatment ideally would include a four‐cell design (PTSD with maltreatment, PTSD without maltreatment, maltreatment without PTSD, no PTSD and no maltreatment) to isolate specific associations of diagnosis versus childhood maltreatment on white matter microstructure (De Bellis et al., 2015). Additionally, because we had few participants who had experienced solely criterion‐A‐type maltreatment or solely noncriterion‐A‐type maltreatment, we were unable to directly compare effect sizes across groups. Our cell sizes are also too small for investigation of sex‐specific effects, which may be present and important. Finally, our maltreatment measure did not include information about the timing and duration of exposure during childhood, so we are unable to further investigate those effects. Future work will employ measures (e.g., MACE: Maltreatment and Chronology of Exposure; Teicher & Parigger, 2015) that allow for assessment of these variables.

## 5 CONCLUSION

To summarize, the present study contributes to a growing body of literature indicating that childhood maltreatment is associated with altered WM microstructure in the ILF, a long association pathway involved in integrating visual information from the posterior cortices with anterior temporal lobe functions including emotion processing. From a clinical perspective, the present work supports the proposition that neglect and emotional abuse can have comparable effects on neurodevelopmental maturation as physical and sexual abuse, highlighting the importance of early detection and intervention with individuals exposed to multiple forms of childhood maltreatment.

## CONFLICT OF INTEREST

The authors have no conflicts of interest to declare in relation to this work.

## Data Availability

The data that support the findings of this study are available from the corresponding author upon reasonable request.
