# Supplementary material for: Childhood maltreatment experiences are associated with altered diffusion in occipito‐temporal white matter pathways
Source: Brain Behav. 2019 Nov 26;10(1):e01485. doi: 10.1002/brb3.1485 (PMC6955831; doi:10.1002/brb3.1485)
Supplement: Supplementary file 1 [file BRB3-10-e01485-s001.docx]

**Supplement 1**

*Materials & Methods: Inclusion/exclusion criteria*

Subjects were eligible to participate in this study if they (1) were 20-50 years of age; (2) were right handed; (3) had a DSM-IV diagnosis consistent with group assignment; and (4) were able to provide informed consent. Groups were matched for education, sex, age, and race/ethnicity. Subjects were ineligible to participate if they (1) had a medical condition that might confound the findings (e.g. HIV, multiple sclerosis); (2) had a history of seizures or a serious head injury followed by a loss of consciousness greater than 5 minutes; (3) had metal implants or other MR exclusions; (4) had claustrophobia; (5) tested positive for psychoactive drug use or pregnancy on a urine screen; (6) had a history of obsessive compulsive disorder, eating disorder, bipolar disorder, intellectual disability, a pervasive developmental disorder or psychotic disorder; (7) currently had panic disorder; and (8) for HC participants, used psychotropic medications. For the PTSD and TENC group, a stable dose of antidepressant medication was acceptable (TENC, N = 1; PTSD, N = 2). Exposure to psychotropic medications aside from antidepressants within 4 weeks prior to participating in the study was exclusionary. Excluding the three medicated participants did not affect the core findings: there was no effect of diagnostic group on ILF FA, F(4,170) = 1.115, p = 0.351, while there was a significant effect of childhood maltreatment, F(2,84) = 3.814, p = 0.026.

HC group participants had no history of any DSM-IV Axis I psychiatric disorder and endorsed no history of trauma exposure meeting DSM-IV Criterion A during the clinical interview. TENC group participants had a history of trauma exposure but never met full DSM-IV criteria for PTSD. PTSD group participants met full DSM-IV criteria for lifetime PTSD and were experiencing symptoms within the past month from at least one of the four symptom clusters of DSM-IV PTSD.

Within the PTSD group, index trauma were diverse and included the following: witnessing unexpected severe injury, sudden life-threatening illness, or death in family member (n = 4); childhood physical or sexual abuse (n = 7); motor vehicle accident (n = 4); physical assault (n = 2); sexual assault (n = 2); terrorist attack (n = 2); civilian combat exposure (n = 1); torture (n = 1); political violence (n = 1); natural disaster (n = 1); mass shooting (n = 1); non-combat military trauma (n = 1); and witnessing assault/suicide/gun violence (n = 5).

*Materials & Methods: Clinical interviews and measures*

In order to assess the presence and nature of past childhood abuse and neglect, the Childhood Trauma Questionnaire (CTQ: Bernstein et al., 2003) was administered to all participants. The CTQ is a 28-item self-report measure. Participants were asked to rate statements about their childhood on a scale from 1 (Never True) to 5 (Very Often True). The CTQ yields five subscale scores each evaluating a specific type of childhood maltreatment – emotional, physical and sexual abuse, and emotional and physical neglect. High scores on the CTQ signify a greater history of childhood maltreatment. The CTQ has been shown to have good criterion-related validity (Bernstein et al., 2003). For the purpose of defining exposure to different types of maltreatment in the sample, exposure to at least moderate severity maltreatment also was coded as present or absent based on cutoffs in the CTQ manual (emotional abuse: 13+, physical abuse: 10+, sexual abuse: 8+, emotional neglect: 15+, physical neglect: 10+).

*Materials & Methods: Neuroimaging parameters*

T1-weighted MPRAGE images were collected using parameters that were optimized for morphometric analysis on Freesurfer software: 128 slices, echo time = 3.31ms, repetition time 2530ms, inversion time= 1100ms, flip angle = 7º, slice thickness = 1.33mm (voxel size: 1.0 x 1.0 x 1.33 mm). DTI parameters were as follows: TR = 8600ms; TE = 90ms; 36 directions at b = 1000 plus 8 b = 0 images; slice thickness = 2.2mm (voxel size: 2.2 mm isotropic); Total acquisition time was 6 min 46 seconds.

*Materials & Methods: Tractography processing*

Structural MPRAGE images were segmented and parcellated using FreeSurfer’s standard pipeline (Dale, Fischl, & Sereno, 1999; Fischl et al., 2002, 2004) and then manually checked for accuracy and edited. Diffusion data underwent preprocessing using TRACULA’s standard pipeline, with steps including eddy-current correction, gradient rotation, brain mask extraction, registration to the T1 scan using bbregister, registration to MNI space, tensor fitting, and computing tract priors from the TRACULA atlas. Within TRACULA, FSL’s bedpost was used to fit a ball-and-stick diffusion model (Behrens et al., 2003). Probability distributions for the ILF and CST were determined by combining information from the ball-and-stick model fit with anatomical priors from the TRACULA atlas. FA images were visually inspected to identify datasets that were visibly corrupted by motion; there were none, so all images were retained. Because of low variance in the portion of slices with drop-out and drop-out score, the total motion index (TMI: Yendiki et al., 2014) in the present dataset was calculated using translation and rotation scores only (as in Olson et al., 2017). TMI was calculated as follows: ((participant’s average volume-by-volume translation – group median translation)/(upper quartile translation-lower quartile translation)) + ((participant’s average volume-by-volume rotation – group median rotation)/(upper quartile rotation-lower quartile rotation)).

*Materials & Methods: Statistical analysis*

MANCOVAs were conducted in SPSS 20 using the multivariate general linear model (GLM) procedure. Right and left ILF FA were entered as dependent variables. Gender was entered as a fixed factor. Age and CTQ total scores were entered as covariates. The model included main effects for gender, age, and CTQ total scores. The intercept was included in the model.

*Results: Childhood Maltreatment Exposure*

Participants endorsing at least moderate maltreatment, by diagnostic group:

|  | PTSD (total N = 32) | TENC (total N = 27) | HC (total N = 34) |
| --- | --- | --- | --- |
| Emotional Abuse | 2 | 8 | 15 |
| Physical Abuse | 1 | 10 | 12 |
| Sexual Abuse | 2 | 7 | 15 |
| Emotional Neglect | 2 | 7 | 15 |
| Physical Neglect | 1 | 3 | 15 |
|  |  |  |  |
| No maltreatment | 6 | 13 | 30 |
| Criterion-A-type-only | 7 | 3 | 1 |
| Non-crit-A-type-only | 5 | 1 | 2 |
| Both | 19 | 10 | 1 |

*Results: ILF FA: Analysis for Sex-specific Effects*

Multivariate models were tested examining the interaction between sex and maltreatment exposure on ILF FA, controlling for main effects of sex and age. There was no significant sex * maltreatment interaction for total maltreatment exposure or non-criterion-A-type exposure. The sex * maltreatment interaction for criterion-A-type maltreatment exposure on ILF FA was significant bilaterally, F (2,86) = 3.982, p = 0.023, though when separate models were run for each sex, the prediction of ILF FA from criterion-A-type maltreatment exposure was non-significant in both males and females.
